# A rapid quality control test to foster the development of genetic control in mosquitoes

**DOI:** 10.1038/s41598-018-34469-6

**Published:** 2018-11-01

**Authors:** Nicole J. Culbert, Fabrizio Balestrino, Ariane Dor, Gustavo S. Herranz, Hanano Yamada, Thomas Wallner, Jérémy Bouyer

**Affiliations:** 1 Insect Pest Control Laboratory, Joint Food and Agriculture Organization of the United Nations/International Atomic Energy Agency Programme of Nuclear Techniques in Food and Agriculture, A-1400 Vienna, Austria; 2 Institute of Integrative Biology, Centre for Genomic Research, University of Liverpool, Liverpool, Merseyside UK; 3 Medical and Veterinary Entomology Department, Centro Agricoltura Ambiente CAA “G. Nicoli”, Via Argini Nord 3351, 40014 Crevalcore, Italy; 4 CONACYT-ECOSUR, Carretera Antiguo Aeropuerto km. 2.5, C.P. 30700 Tapachula, Chiapas Mexico; 5 Technical School of Design, Architecture and Engineering, University CEU Cardenal Herrera, 46115 Calle San bartolomé 55 Alfara del Patriarca, Valencia, Spain; 6 CIRAD, UMR ASTRE CIRAD-INRA ≪AnimalS, health, Territories, Risks and Ecosystems≫, Campus international de Baillarguet, 34398 Montpellier, France

**Keywords:** Environmental biotechnology, Invasive species

## Abstract

Vector-borne diseases are responsible for more than one million deaths per year. Alternative methods of mosquito control to insecticides such as genetic control techniques are thus urgently needed. In genetic techniques involving the release of sterile insects, it is critical to release insects of high quality. Sterile males must be able to disperse, survive and compete with wild males in order to inseminate wild females. There is currently no standardized, fast-processing method to assess mosquito male quality. Since male competitiveness is linked to their ability to fly, we developed a flight test device that aimed to measure the quality of sterile male mosquitoes via their capacity to escape a series of flight tubes within two hours and compared it to two other reference methods (survival rate and mating propensity). This comparison was achieved in three different stress treatment settings usually encountered when applying the sterile insect technique, i.e. irradiation, chilling and compaction. In all treatments, survival and insemination rates could be predicted by the results of a flight test, with over 80% of the inertia predicted. This novel tool could become a standardised quality control method to evaluate cumulative stress throughout the processes related to genetic control of mosquitoes.

## Introduction

Vector-borne diseases account for 17% of infectious diseases leading to more than one million deaths each year^1^. The toxicity and ecotoxicity of insecticides together with the spread of resistances to pyrethroids urge the development of alternative mosquito control methods, particularly against *Aedes* vectors. In their global vector control response 2017–2030, the World Health Organization (WHO) indicates the urgent need for alternatives^2^. Many new mosquito control methods are thus being tested^1^, among which genetic control shows promises^3^.

The sterile insect technique (SIT) is a birth control method based on repeatedly releasing large numbers of sterile male insects to reduce the reproduction in a target population of the same species^4^. For over six decades, the SIT has been implemented globally through area-wide integrated pest management programs (AW-IPM) to suppress, contain, prevent or even eliminate insect pests of agricultural and medical/veterinary importance, such as fruit flies^5^, screwworms^6^ and tsetse flies^7^. Despite promising results from initial pilot studies^8^, research on mosquito SIT dwindled. However, with current control methods not allowing sustainable management of *Aedes* vectors, together with a lack of effective vaccines, an interest in SIT as a new tool within mosquito AW-IPM programs has been reignited^1^.

Reaching the operational level in any SIT program is no easy feat. Establishing mass rearing techniques, standardising irradiation methods, developing a stable sexing system and developing release technology are, to name but a few, all essential criteria which must be fully understood in order to achieve a successful program. Furthermore, in AW-IPM approaches that contain an SIT component, the quality of the sterile insects remains one of the fundamental criteria for a successful program^9^. Sterile male insects have one goal and that is to mate with wild females and induce sterility within the target population. Poor quality males may have damaged wings, missing limbs or a shortened lifespan and thus will be unable to compete with wild males in the field. Maintaining high quality management of sterile males is crucial to counteract the reduced filed performance that arises from the stress-related impacts of biological or operational attributes such as mass rearing, irradiation, handling, transport and release processes^8^.

For many years, SIT was seen as a numbers game and if a program exhibited signs of failure, the thought process was simply to release *more* insects to compensate^9^. This was due to an absence of a means to evaluate the effectiveness of mass reared sterile insects and interactions with their wild counterparts, with quality control tools only coming into practice latter^10^. Today, quality control systems are well established for the production and release of various species of sterile insects^11,12^. Insect quality must be routinely assessed and if necessary, improved, via a series of bioassays during the production process within a mass rearing facility. Life history parameters such as egg hatch rate, developmental time, pupal size, sex ratio, adult emergence percentage, longevity are regularly measured. Furthermore, the quality of sterile insects post-release must be assured by evaluating flight ability, dispersal capability, sperm transfer, mating propensity and competitiveness^9^. There is a distinct lack of quality control methods to evaluate the quality of sterile male mosquitoes. Current systems routinely involve arduous laboratory, semi-field and field tests, such as mark-release-recapture (MRR) studies to ascertain dispersal, longevity and competitiveness^13,14^. Thus, the demand for quick, cost-effective quality control tools is increasing.

Insect flight ability is known to be a direct, reliable marker of insect quality^15,16^. Tools such as flight mills^17^, already exist for assessing mosquito flight ability but would simply not be practical for routine use in a mass rearing facility or field site. However, for sterile fruit flies, tsetse flies and moths, flight cylinders, normally composed of PVC tubes are used to gauge flight ability, which has been demonstrated to be a good proxy of mating competitiveness^18,19^. Flight cylinders are inexpensive, quick and portable, enabling routine quality tests to be carried out both during the production chain and post-release. Recently, new quality control devices have been designed to infer the survival and mating capacity of radio-sterilized *Aedes albopictus* males through the observation of flight capacity of newly emerged adults from individual pupae^20^. This test was however time consuming (48 H to 72 H) and did not allow measuring the impact of various treatments to which adults are subjected from their production to their release. In order to improve the practicality, manoeuvrability and response time of the flight organ devices, a new flight cylinder device capable to test batches of 100 adults directly within a two hour period without introducing them at pupal stage was proposed. We present the results of a series of validation tests during which *Ae*. *albopictus* and *Ae*. *aegypti* adult mosquitoes were subject to varying levels of stress treatments which are known to affect mosquito quality, including irradiation, chilling and compaction^9^. Flight ability was subsequently measured and compared to the results of mating capacity and survival which were measured as reference tests. The goal of this study was thus to validate a novel flight test device as a quality control tool for the genetic control of insects.

## Results

### Impact of treatments on survival and insemination rates

**Figure 1:**
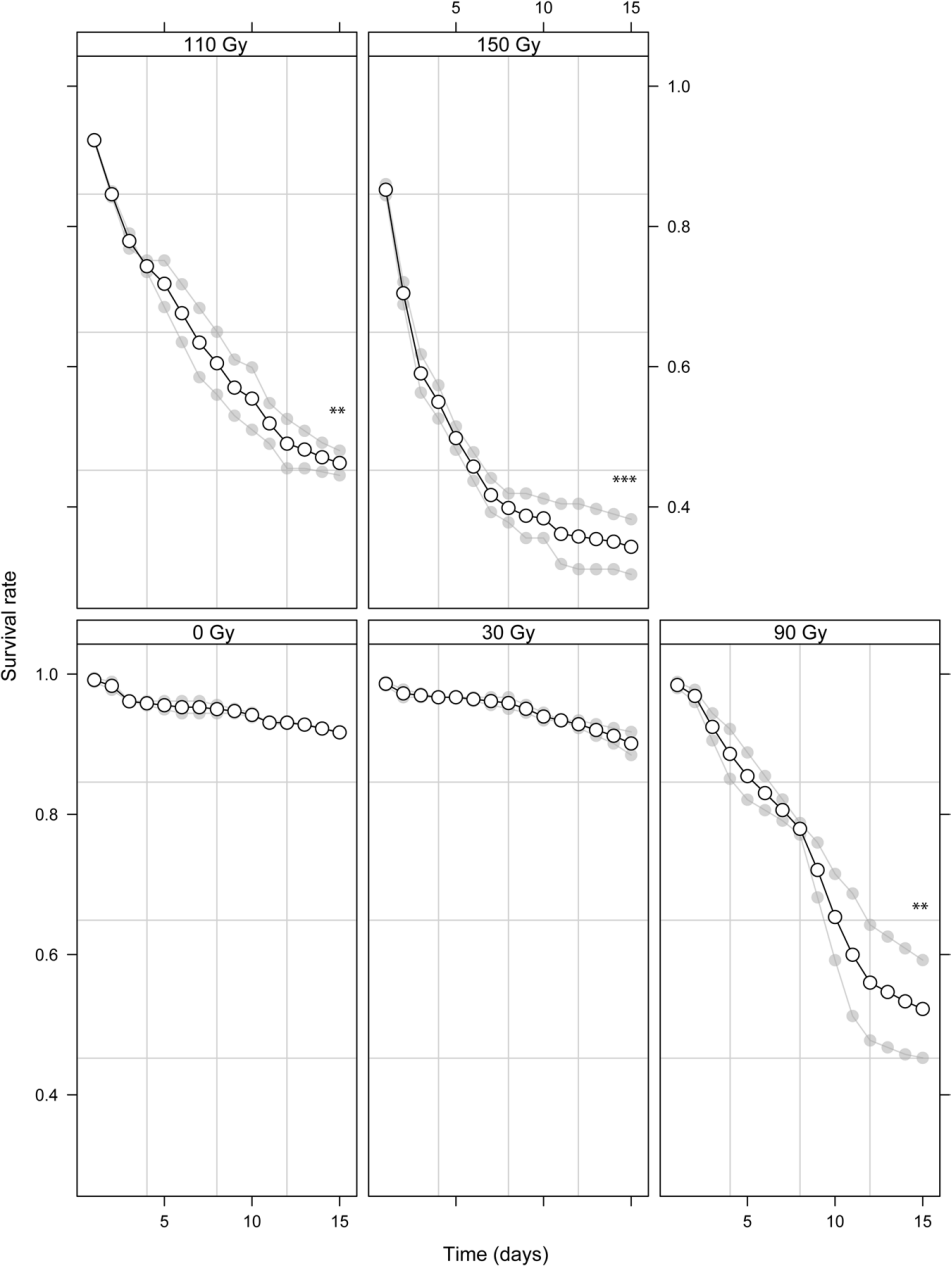
Survival rates of male *Aedes aegypti* exposed to various irradiation doses over a period of 15 days. Significant differences between treatment groups (30, 90, 110 and 150 Gy) and the control group (no irradiation) are indicated (*p < 0.005, **p < 0.01; ***p < 0.001). Individual values of the repeats are indicated in light grey and mean values as a solid line.

**Figure 2:**
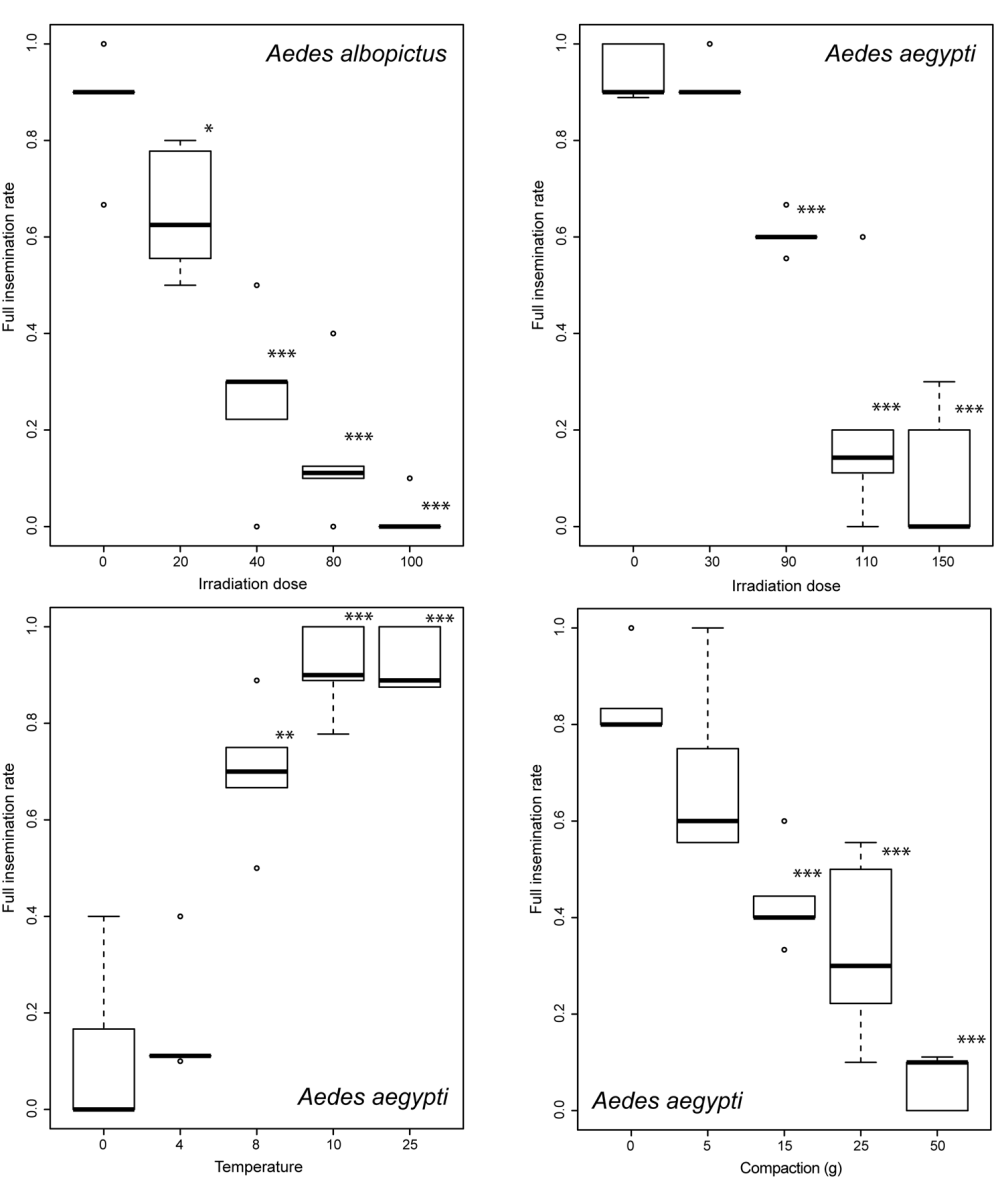
Full insemination rates of male *Aedes* mosquitoes exposed to various treatments. The top panels present the impact of various irradiation doses on *Aedes albopictus* (left) and *Ae*. *aegypti* (right). The bottom panels present the impact of chilling (left) and compaction (right) on *Ae*. *aegypti*. Boxplots present the median value and the quartiles, horizontal bars the 95% percentiles and dots the minimal and maximal values. Significant differences between treatment groups and the control group are indicated (*p < 0.005, ** p < 0.01; *** p < 0.001).

Irradiation reduced survival significantly at a dose equal to or superior than 90 Gy in *Aedes aegypti* (Fig. 1 and Table S1, p < 10e-3) and 40 Gy in *Ae*. *albopictus* (Fig. S1 and Table S2, p < 10e-3). It also reduced the full insemination rate significantly starting from 90 and 20 Gy in *Aedes aegypti* (Table S3, p < 10e-3) and *Ae*. *albopictus* (Table S4, p = 0.01) respectively (Fig. 2). The insemination rate was less sensitive than the full insemination rate, with a significant decrease in *Ae*. *albopictus* only, commencing from 40 Gy (Fig. S2 and Table S5, p = 0.02).

Considering the impact of chilling on male quality in *Ae*. *aegypti*, the survival rate was significantly reduced only at a temperature of 0 °C (Fig. S3 and Table S6, p < 10e-3) while the full insemination rate already began declining from exposure to 8 °C (Table S7, p < 0.01). Again, the insemination rate appeared less sensitive than these two aforementioned parameters (Fig. S2).

Finally, compaction significantly impacted the survival of *Ae*. *aegypti* from a weight of 5 g (0.25 g/cm^2^) onwards (Fig. S4 and Table S8, p < 0.05), illustrating how fragile this insect species is. The full insemination rate was reduced only when the weight exceeded 15 g (Table S9, p < 10e-3) and the insemination rate was again less sensitive as seen with irradiation and chilling data above (Fig. S2).

### Flight test device

**Figure 3:**
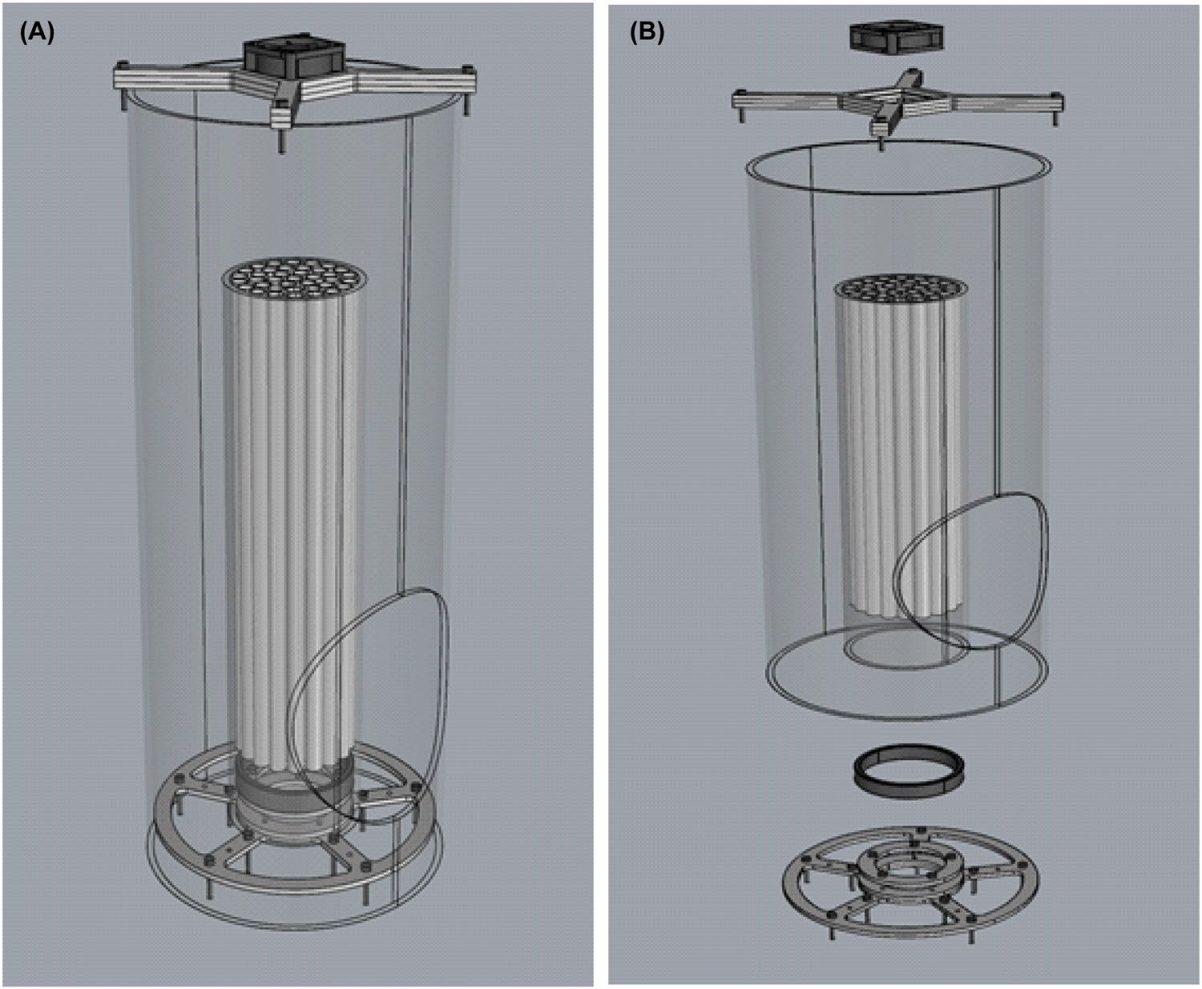
The flight test device (FTD). A complete overview of the FTD in panel A. The placing of each component can be depicted from panel B.

Flight ability was measured by aspirating a sample of 100 adult male *Aedes aegypti* or *albopictus* into one of the flight test devices (FTD) via a small 1 cm hole at the bottom of the device (see Fig. 3). The mosquitoes are then within a confined space of 1 cm in height and thus their natural instinct is to fly upwards via one of the 40 flight tubes (25 cm high, inside diameter of 8 mm) and out into the large, containment tube. After filling the FTD with mosquitoes, one small pellet of BG lure (Biogents, Regensburg, Germany) is placed on the top, directly underneath a 12 V fan that is then switched on. The fan speed is 6000 revolutions per minute (rpm) capable of generating an airflow of 11.9 m^3^/hour. After two hours, the fan is stopped and the experiment is classed as finished. The FTD is then taken to a cold room (4 ± 1 °C) and after 5–10 minutes when the mosquitoes are immobile, the number of adults still remaining within the flight tubes or underneath them and those who successfully escaped are counted. The number of escaped males is divided by the total number of males, thus generating an escape rate.

### Impact of treatments on flight ability

**Figure 4:**
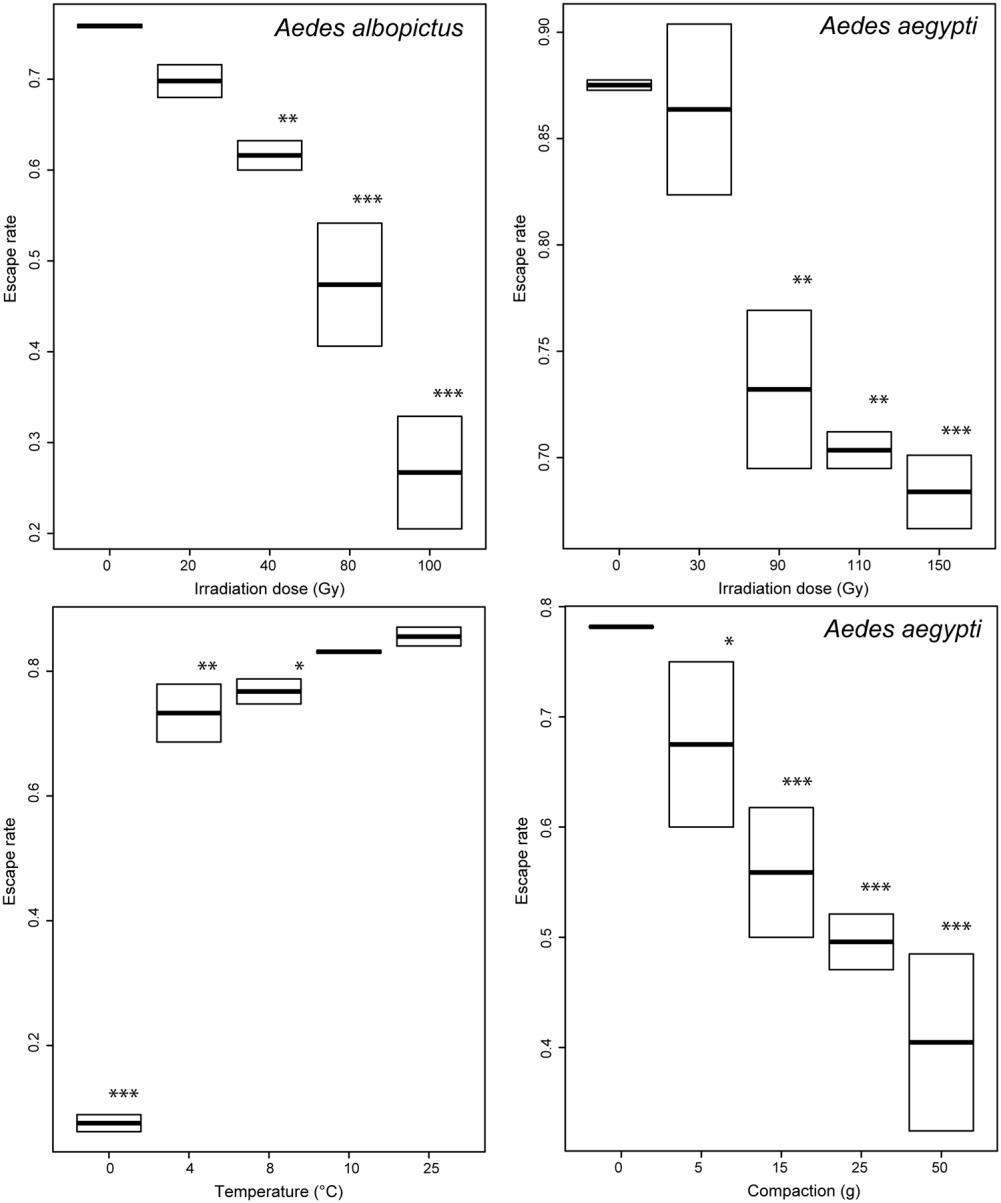
Escape rates of male *Aedes* mosquitoes exposed to various treatments. The top panels present the impact of various irradiation doses on *Aedes albopictus* (left) and *Ae*. *aegypti* (right). The bottom panels present the impact of chilling (left) and compaction (right) on *Ae*. *aegypti*. Boxplots present the median value and the quartiles, horizontal bars the 95% percentiles and dots the minimal and maximal values. Significant differences between treatment groups and the control group are indicated (*p < 0.005, **p < 0.01; ***p < 0.001).

Flight ability measured as described upon overall appeared as an excellent quality control parameter since it was sensitive to all treatments (Fig. 4, Tables S10–S13). It predicted accurately the different thresholds impacting other parameters (Table 1), explaining 78 to 92% of the variance of survival rates, 62 to 95% of the variance of insemination rates and 53 to 86% of the variance of full insemination rates. It was interesting to see that the survival rate was more sensitive to the compaction treatment than the full insemination rate whereas the contrary was observed for chilling. Flight ability was in both cases as sensitive as the most sensitive of the two others, with the only exception of irradiation dose in *Ae*. *albopictus*, which gave a significant reduction of the full insemination rate at 20 Gy already whereas it reduced the flight capacity starting from 40 Gy (Table S10, p = 0.007).

**Table 1:** Use of the male escape rates from the flight organ to predict adult male quality parameters.

Species	Treatment	First significant impact on escape rate	First significant impact on survival rate at day 15	First significant impact on insemination rate	First significant impact on full insemination rate
*Aedes aegypti*	Irradiation	90 Gy	90 Gy (0.819)	NA (0.951)	90 Gy (0.840)
	Chilling	8 °C	0 °C (0.802)	NA (0.616)	8 °C (0.532)
	Compaction	5 g	5 g (0.776)	NA (0.879)	15 g (0.812)
*Aedes albopictus*	Irradiation	40 Gy	40 Gy (0.918)	40 Gy (0.790)	20 Gy (0.859)

The first values of the different treatments significantly impacting each male quality indicator are presented. The values in brackets correspond to the proportion of explained variance (r-square), used as a model quality indicator, based on a linear mixed-effect model where the response variable (survival, insemination and full insemination rates) is predicted using the escape rate as a fix effect and the repeats as random effects. All p-values of the predictions were below 0.001. Survival was quantified by removing and counting dead individuals from both control and experimental cages daily for a period of 15 days. Mating propensity was calculated by measuring the number of virgin females (n = 10) a single control or post stress treatment male could successfully inseminate during a period of 5 days. Females were scored as inseminated or fully inseminated if one or two or more spermatheca contained sperm respectively. NAs correspond to cases in which models did not converge, mostly because the insemination rate was 1 in some of the treatments.

## Discussion

Inducing sterility in insects is most commonly achieved via ionizing radiation. However, it has been repeatedly reported to impact the subsequent survival and quality of the insect^8^. Thus, the balance between quality and sterility is a delicate one. Administering too low a dose will cause insects to retain high levels of fertility whilst too high a dose will severely impact the field competitiveness of the insect. High irradiation doses increase the level of somatic damage and thus decrease the quality of the insect which will in turn exhibit reduced mating capacity, flight capacity and longevity. Releasing poor quality insects will decrease the effectiveness of an SIT program, make it more costly and thus require more insects to be released, or the overflooding ratio to be increased^5^. It is recommended to select a lower irradiation dose and release a more competitive insect when confronted with this trade off ^21^. *Ae*. *albopictus* has been shown to be partially and fully sterile at 35 and 40 Gy respectively whilst still equally as competitive as non-irradiated controls^14,22,23^ thus we chose our irradiation doses based around this knowledge. On the other hand, an absence of irradiation literature regarding *Ae*. *aegypti* meant that the doses selected were based on personal communications within the IPCL (partially sterilising dose of 90 Gy). Surprisingly, we noted that our standard irradiation doses of 40 and 90 Gy for *Ae*. *albopictus* and *Ae*. *aegypti* respectively caused significant decreases in quality in all measured parameters. This is in contrast to previous findings on competitiveness measured in semi-field experiments which might indicate that flight ability is even more sensitive than the latter. A limit of our study is that flight ability has not yet been compared to semi-field and field competitiveness although this is planned in the next future and our preliminary results seem very promising. We thus advise member states and research institutes willing to use our technology for routine monitoring of the quality of their sterile males to first establish a reference comparison between flight ability of their strain and competitiveness in their particular environmental settings.

In current SIT programs, insects are routinely exposed to chilling in order to immobilise them to facilitate their handling and eventual field release, such as Mediterranean fruit flies (*Ceratitis capitata*) which are maintained at 4 °C for up to 3 hours prior to an aerial release^19^. In contrast to other species of sterile insects, there is a distinct gap in the literature regarding the handling, transport and release of sterile male mosquitoes. Based upon a recent publication^24^, and following preliminary trials within our laboratory with *Aedes aegypti*, we were able to determine a range of immobilisation temperatures for our chilling stress treatment. We predicted that when male *aegypti* were chilled at 8 and 10 °C, they would be of equal quality to controls in contrast to those exposed to 4 and 0 °C. Interestingly, our results indicated that only exposure to the lowest chilling temperature, 0 °C, significantly decreased their survival 15 days after exposure, a similar result to what was found in *Anopheles arabiensis* which only exhibited a significantly reduced survival when exposed to 2 °C which was also the lowest chilling temperature within the study^24^. However a significant decrease in flight ability was noted after chilling at 8 °C. This is similar to what has been noted in recently emerged tsetse flies after the shipping of chilled pupae at 8 °C for up to 72 hours^18^. This is however in contrast to what has been observed in sterile fruit flies where chilling only has a significant impact on flight ability and mating competitiveness when flies are maintained in crowded conditions prior to being chilled for between 0 and 3 hours^19^. These results emphasise how chilling can impact sterile insects differently according to species, the duration of chilling or the conditions prior to chilling i.e. crowding, in addition to highlighting the importance of routine quality control checks via devices such as a flight cylinder. It may be of value to conduct tests within the FTD following chilling at different temperatures for varying lengths of time and perhaps densities to try to disentangle the effects of each parameter with regard to male quality and to ascertain if a synergetic effect arises from independent parameters. It would also be useful to evaluate if chilling may cause a reduction of sperm mobility through cellular or physiological damages, and result in the reduced rate of full insemination observed for temperatures of 8 °C and below. Actually, the full insemination rate was proportionally more reduced than the flight ability at 4 °C for example.

Unlike in SIT programs involving tsetse or fruit flies, mosquitoes will be transported to release sites in their adult phase as opposed to pupal. Dealing with the fragility of such an insect poses unique questions. One grey area has been the maximum capacity of adult mosquitoes that can be stored and how tolerant they are to compaction. We suspected that immobile males would become damaged if the load above them was too high. Our results confirmed that even a weight of 5 g (0.25 g/cm^2^), was enough to significantly decrease longevity, which will be of great value when designing transportation boxes or cassettes for adults in addition to the maximum capacity that can be maintained within each box. Overcrowding was found to impose a synergetic effect on fruit flies when flies were held immobile, one which can be reversed by maintaining flies at lower densities. Independently, chilling and crowding did not cause any significant effect upon mating success or flight ability^19^.

As mosquito SIT moves closer to an operational level the necessity to accurately determine the quality of sterile males at every point in the production chain and afterwards grows. Our FTD allows a sample of 100 mosquitoes to be sampled in one device, which is significantly higher than current flight mills where a maximum of 16 insects can be sampled at any given time^25^. Moreover, we are currently using ten devices simultaneously but due to the low cost, ease of use and few parts necessary to construct the FTD, there is a limitless possibility of how many insects from various cohorts or stages of the mass rearing procedure that could be tested at the same time. Finally, our FTD requires only two hours to measure flight ability whereas measuring insemination rates requires 6 days, survival and semi-field competitiveness 15 days and the former fight device at least 48H^20^. Our FTD will thus be a useful and effective tool for monitoring and providing feedback on the quality of sterile male mosquitoes during the production, handling and release phases of a control programme that comprise an SIT component. All technical drawings of the FTD allowing producing it are available on our website (http://www-naweb.iaea.org/nafa/ipc/public/manuals-ipc.html). Our results may also be useful for all strategies based on genetic control that depend on the release of sexually competitive mosquitoes, including *Wolbachia-*infected mosquitoes^26,27^, RIDL^28,29^ or gene drive^30,31^.

## Methods

### Mosquito Colony Rearing

The strains of *Ae*. *aegypti* and *Ae*. *albopictus* used in all experiments originated from Juazeiro, Brazil and Rimini, Italy, respectively. They were transferred to the Food and Agricultural Organisation/International Atomic Energy Agency (FAO/IAEA) Insect Pest Control Laboratory (IPCL) in Seibersdorf, Austria by Biofabrica Moscamed, Brazil and Centro Agricoltura Ambiente “G.Nicoli” (CAA), Italy respectively. They are maintained in climate controlled insectary (temperature 27 ± 1 °C, relative humidity 70 ± 10%, photoperiod 12:12, with two one-hour twilight periods simulating dawn and dusk) as was previously described by (24). For all experiments, larvae were reared in plastic trays (40 × 29 × 8 cm) containing 1 litre of deionized water at a density of approximately 3000 first instar (L_1_) per tray and were provided with the IAEA-2 diet following the protocol described in^32–34^.

### Irradiation Procedure and Experimental Design

Pupae were separated from larvae and sexed mechanically (John W. Hock Co., Gainesville, FL) prior to further examination under a stereomicroscope, ensuring pure batches of males and females. Male pupae were irradiated at 36 ± 4 hours in batches 150 inside a self-contained ^60^Co Gamma Cell 220. Dose accuracy was measured with a dosimetry system using Gafchromic MD film. A range of irradiation doses were selected for each species, including the values necessary to induce full sterility and then beyond to severely reduce the quality of the adults. With 0 Gy representing the controls for each species, 30, 90, 110 and 150 Gy and 20, 40, 80 and 100 Gy were chosen for *Ae*. *aegypti* and *Ae*. *albopictus* respectively.

Adults were maintained in standard plastic cages (30 × 30 × 30 cm – Bugdorm, Taiwan) with continued access to a 10% sucrose solution until day 3 when experiments were performed. Mosquito maintenance and the age of the adults when all described experiments were performed was chosen to reflect what would occur in a mass rearing facility prior to a release of sterile males. There were two replicates for each stress treatment in addition to two control samples for each experiment performed.

### Chilling Procedure and Experimental Design

As with irradiation, a range of chilling temperatures were selected for *Aedes aegypti* that were known to be within a tolerable limit and others were chosen with the aim that they would impact quality following exposure. When age 3 days, batches of 250 adult males were immobilised and held for two hours at 0, 4, 8 or 10 °C with control males left in insectary conditions (27 ± 1 °C).

### Compaction Procedure and Experimental Design

Batches of 250 adult male *Ae*. *aegypti* were immobilised at 10 °C, a temperature known not to impact their quality, for a period of two hours. During this period, they were subject to various levels of compaction by adding 0, 5, 15, 25 or 50 g weights, corresponding to 0, 0.25, 0.76, 1.27 and 2.55 g/cm^2^ respectively. Cumin seeds were wrapped in mesh and sealed with an elastic band to serve as a substitute for mosquitoes during this experiment. The morphological properties and weights of various substitute particles including rice, poppy, anise, fennel and cumin seeds were analysed previously with cumin seeds found to best match the weight and characteristics of adult mosquitoes, hence their selection for this experiment.

### Assessing Survival Rate and Mating Propensity as a Measure of Quality

The survival rate and mating capacity of males under each of the aforementioned stress treatments (irradiation, chilling or compaction) were measured with the aim to link these known quality parameters with their flight ability post stress treatment. The survival rate was quantified by removing and counting dead individuals from both control and experimental cages daily for a period of 15 days (2 repetitions per treatment). The number of adults remaining for longevity assessment (N) varied slightly between experiments. For the irradiation experiments, N varied from 114–197 and 64–151 for *Aedes aegypti* and *albopictus* respectively. For the temperature and compaction experiments, N varied from 109–149 and 118–173 respectively for *Aedes aegypti*. Mating propensity was calculated by measuring the number of virgin females a single control or post stress treatment male could successfully inseminate during a period of 5 days. A single adult male mosquito, from each batch of 250 controls and treatment cages, was transferred to a small cage (30 × 30 × 30 cm) containing 10 virgin females from the same cohort. There were 5 repetitions for all treatments and the control for mating capacity tests and all adults were allowed continued access to a 10% sucrose solution. Afterwards, each female was dissected and all 3 spermathecae removed to check for the presence or absence of sperm under a stereomicroscope. Females were scored as inseminated and fully inseminated if at least one and two or more spermatheca contained sperm respectively.

### Flight Test Device and Experimental Procedure

A flight test device (FTD), which aims to evaluate the flight ability of an adult mosquito, was created after experimental testing (*SI Methods*). The FTD consists of a series of 40 transparent acrylic plastic (Polymethyl methacrylate - PMAA) flight tubes, surrounded by a larger PMAA tube. The first two series of tubes are housed within a third PMAA tube of greater size which serves as a containment box after mosquitoes escape the flight tubes (see *SI Methods* for complete dimensions).

Mosquitoes were blown into the FTD via a mouth aspirator and given a period of 2 hours to escape. Afterwards, the number of adults that remained at the base of the flight tubes and those that have escaped were counted. Flight ability was calculated by dividing the number of adults which escaped by the total number which entered the flight tube. For this test 2 repetitions were conducted for each treatment.

### Statistical Analysis

Binomial linear mixed effect models were used to analyze the impact of the various treatments on survival rates at day fifteen, insemination rates, full insemination rates and escape rates from the flight test device (response variables). The treatment regimens for irradiation, chilling and compaction were then used as fixed effects and the repetitions as random effects. The significance of fixed effects was tested using the likelihood ratio test^35,36^. We also used binomial linear mixed effect models to analyze how the escape rate could explain the three other quality control parameters (survival rates at day fifteen, insemination rates, full insemination rates). To do so, the quality control parameters were used as response variables and the escape rate as a fix effect. The R^2^ (coefficient of determination) was then used to describe the proportion of variance explained by the model between the observed and predicted values^37,38^. Fixed-effects coefficients of all models and their corresponding p-values are reported in Tables S1 to S13, except the ones that did not converge, corresponding to NAs in Table 1.

## Electronic supplementary material

Supplementary information

Raw dataset

## Data Availability

All raw data are available as a Supplementary File.
