# Author Correction: A rapid quality control test to foster the development of genetic control in mosquitoes

**DOI:** 10.1038/s41598-019-44071-z

**Published:** 2019-06-05

**Authors:** Nicole J. Culbert, Fabrizio Balestrino, Ariane Dor, Gustavo S. Herranz, Hanano Yamada, Thomas Wallner, Jérémy Bouyer

**Affiliations:** 1 Insect Pest Control Laboratory, Joint Food and Agriculture Organization of the United Nations/International Atomic Energy Agency Programme of Nuclear Techniques in Food and Agriculture, A-1400 Vienna, Austria; 2 Institute of Integrative Biology, Centre for Genomic Research, University of Liverpool, Liverpool, Merseyside, UK; 3 Medical and Veterinary Entomology Department, Centro Agricoltura Ambiente CAA “G. Nicoli”, Via Argini Nord 3351, 40014 Crevalcore, Italy; 4 CONACYT-ECOSUR, Carretera Antiguo Aeropuerto km. 2.5, C.P, 30700 Tapachula, Chiapas Mexico; 5 Technical School of Design, Architecture and Engineering, University CEU Cardenal Herrera, 46115, Calle San bartolomé 55 Alfara del Patriarca, Valencia, Spain; 6 CIRAD, UMR ASTRE CIRAD-INRA ≪AnimalS, health, Territories, Risks and Ecosystems≫, Campus international de Baillarguet, 34398 Montpellier, France

Correction to: *Scientific Reports* 10.1038/s41598-018-34469-6, published online 01 November 2018

The original version of this Article omitted an affiliation for Nicole J. Culbert. The correct affiliations for Nicole J. Culbert are listed below:

Insect Pest Control Laboratory, Joint Food and Agriculture Organization of the United Nations/International Atomic Energy Agency Programme of Nuclear Techniques in Food and Agriculture, A-1400, Vienna, Austria.

Institute of Integrative Biology, Centre for Genomic Research, University of Liverpool, Liverpool, Merseyside, UK.

This has now been corrected in the HTML and PDF versions of this Article, and in the accompanying Supplementary Material.

