# Supplementary material for: A rapid quality control test to foster the development of genetic control in mosquitoes
Source: Sci Rep. 2018 Nov 1;8:16179. doi: 10.1038/s41598-018-34469-6 (PMC6212531; doi:10.1038/s41598-018-34469-6)
Supplement: Supplementary file 1 — Supplementary information [file 41598_2018_34469_MOESM1_ESM.docx]

**Title:** A rapid quality control test to foster the development of genetic control in mosquitoes

**Authors: Nicole J. Culbert^1,2^, Fabrizio Balestrino^3^, Ariane Dor^4^, Gustavo S. Herranz^5^, Hanano Yamada^1^, Thomas Wallner^1^, and Jérémy Bouyer^1,6*^**

^1^Insect Pest Control Laboratory, Joint Food and Agriculture Organization of the United Nations/International Atomic Energy Agency Programme of Nuclear Techniques in Food and Agriculture, A-1400 Vienna, Austria

^2^ Institute of Integrative Biology, Centre for Genomic Research, University of Liverpool, Liverpool, Merseyside, UK.

^3^Medical and Veterinary Entomology Department, Centro Agricoltura Ambiente CAA "G. Nicoli”, Via Argini Nord 3351, 40014 Crevalcore, Italy

^4^CONACYT-ECOSUR, Carretera Antiguo Aeropuerto km. 2.5, C.P. 30700, Tapachula, Chiapas, Mexico

^5^Technical School of Design, Architecture and Engineering, University CEU Cardenal Herrera, 46115 Calle San bartolomé 55 Alfara del Patriarca Valencia, Spain

^6^CIRAD, UMR ASTRE CIRAD-INRA « AnimalS, health, Territories, Risks and Ecosystems », Campus international de Baillarguet, 34398 Montpellier cedex 05, France

* [j.bouyer@iaea.org](mailto:j.bouyer@iaea.org)

**Supporting Information**

**SI Methods**

**Flight Test Device and Experimental Procedure. *Dimensions.*** The flight test device (FTD) consists of a series of 40 transparent acrylic plastic (Polymethyl methacrylate - PMAA). The height = 25cm, external diameter = 1cm, internal diameter = 0.8cm, wall thickness = 0.1cm. These tubes are encased within a larger PMAA tube (height = 29cm, external diameter = 8cm, internal diameter = 7.2cm, wall thickness = 0.4cm) and gaps between tubes are sealed with silicone gel. There is a 4cm gap at the bottom of this tube with a hole (1 cm diameter) half way up which serves as an entry point for the mosquitoes to be aspirated into the flight tubes. The flight tubes and outer casing are housed within a third, larger PMAA tube (height = 40cm, external diameter = 18cm, internal diameter = 17.2cm, wall thickness = 0.4cm) which contains the mosquitoes after they have successfully exited the flight tubes. This containment tube is closed entirely at the top with a solid mesh. The bottom of the tube has a mesh sleeve where the middle sized PMAA tube containing the flight tubes is inserted and removed. Mosquitoes are aspirated into the base of the PMAA tube to begin a flight ability test via an opening in the middle of the containment tube. This allows them to be directly aspirated into the base to begin the test. This hole is 10 cm in diameter and is surrounded by a mesh sleeve which can be tied closed. The middle-sized tube containing the flight tubes sits on a plastic base to allow sufficient ventilation. Once placed upon the base, the hole where the mosquitoes enter is raised up blocking the entry point and preventing them from escaping. Above the containment tube is a plastic fan holder which holds a 12 V fan in place. Before beginning a flight test, one blue pellet of BG lure is placed directly underneath the fan prior to it being switched on. The fan is connected directly to a power pack. The lure serves as an attractant to encourage the mosquitoes to exit the tubes.

**Flight Test Device and Experimental Procedure. *Prior Laboratory Testing.*** The final design of the FTD was reached after lengthy laboratory testing. Several configurations were tried and tested to develop the optimum dimensions and parameters for the final design. A black cover was used to hide the bottom of the flight test tubes in one experiment. This idea was rejected as the black coloration served as a resting place and the mosquitoes failed to fly out of the flight tubes. The FTD was tested with and without 5 and 12 V dc fans. Initially tests were carried out with the 5V fan and it did not increase the escape rate of the mosquitoes. However, when a 12V fan was tested, it increased the escape rate, most likely due to the greater air flow and thus was included in the final design. Experiments were carried out with and without BG lure pellets. Initially, the BG lure was not thought to encourage the mosquitoes to exit the flight tubes. This may be due to the experiments being conducted with the 5V fan. When tested with the 12V, the BG lure was found to increase the escape rate. The orientation of the device (horizontal, vertical or 45° angle) was investigated. Horizontal positioning of the FTD enabled the mosquitoes to walk out of the flight tubes instead of flying and thus was rejected. When positioned at a 45° angle, it was again found to be too easy for the mosquitoes to escape the flight tubes, thus a vertical position was chosen. Experiments were conducted to ascertain the optimum length of time allowed within the FTD. Initial results showed a significant increase in escape rate when allowing 18 hours in comparison to 15, 30, 60 and 120 minutes. However, when using the 12V fan and BG lure it was found that 120 minutes was optimum and allowing extra time did not significantly improve the escape rate. Lastly, initial tests were conducted with flight tubes of 40 cm in height (outside diameter 10 mm, inside diameter 8 mm). This was later revised down to 25 cm and found to improve the escape rate significantly.

**SI Figures:**


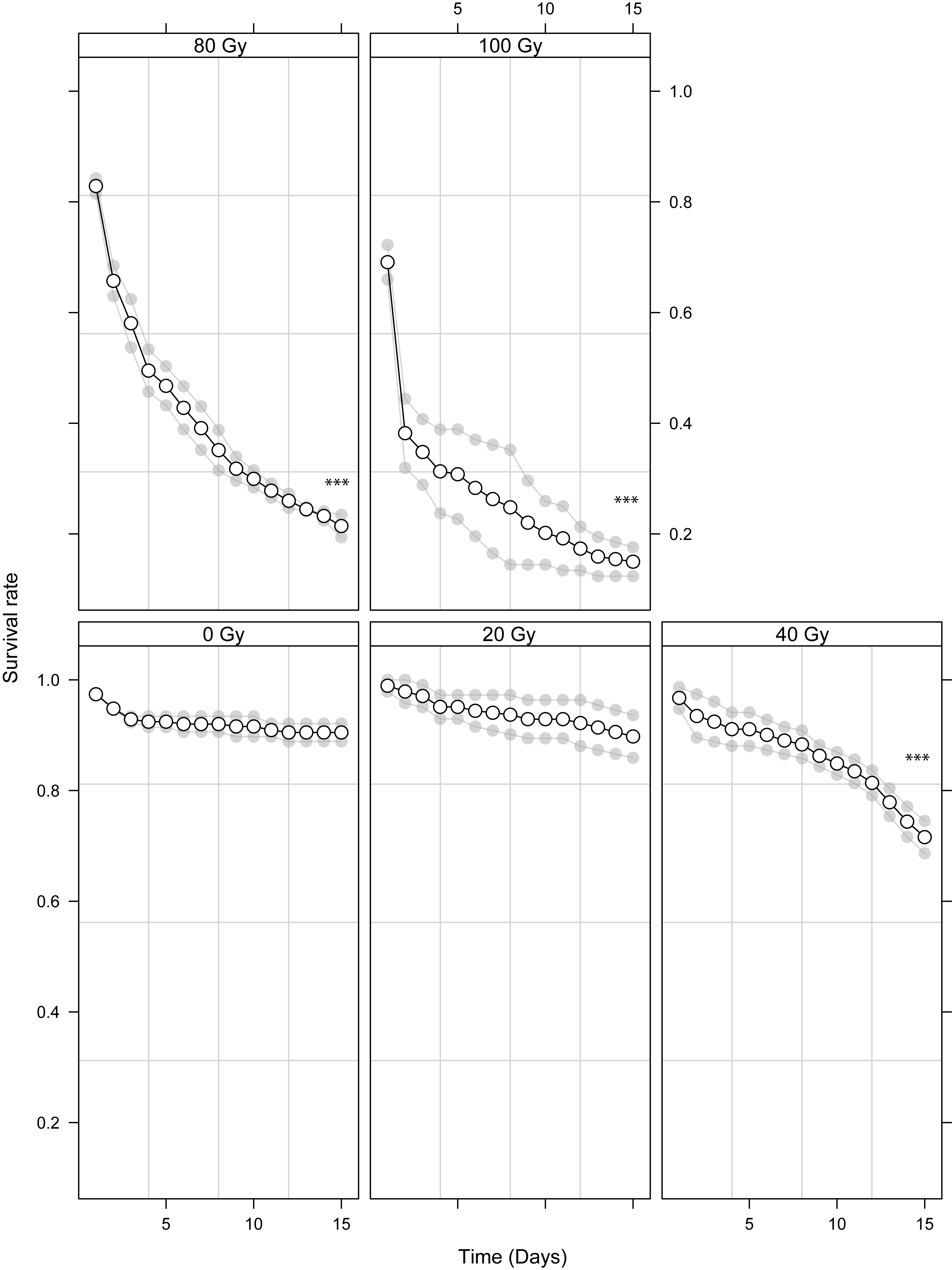


**Fig. S1.** Survival rates of male *Aedes albopictus* exposed to various irradiation doses over a period of 15 days. Significant differences between treatment groups (20, 40, 80 and 100 Gy) and the control group (no irradiation) are indicated (* p<0.005, ** p <.01; *** p < 0.001).. Individual values of the repeats are indicated in light grey and mean values as a solid line.


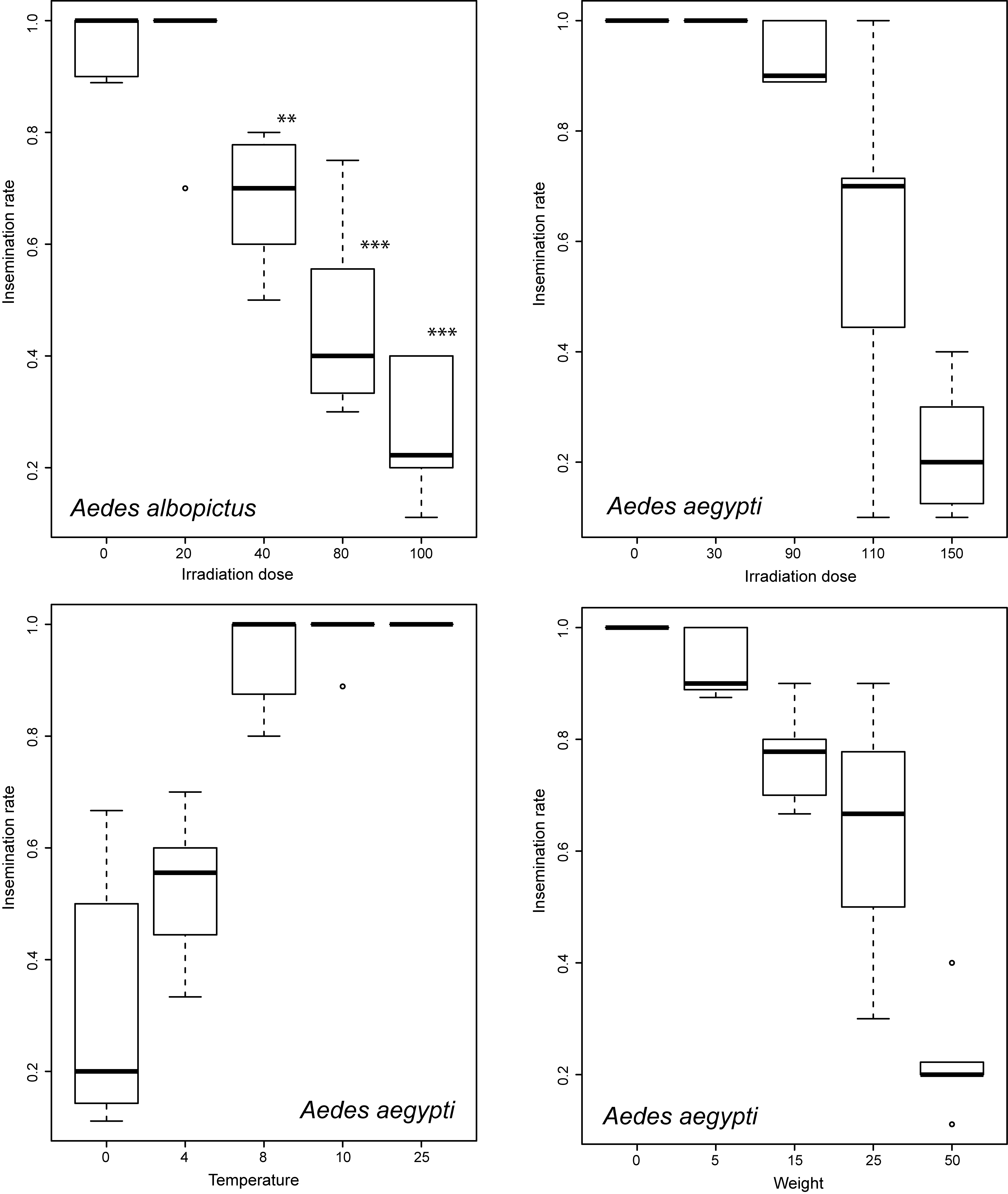


**Fig. S2.** Insemination rates of male *Aedes* mosquitoes exposed to various treatments**.** The top panels present the impact of various irradiation doses on *Aedes albopictus* (left) and *Ae. aegypti* (right). The bottom panels present the impact of chilling (left) and compaction (right) on *Ae. aegypti.* Boxplots present the median value and the quartiles, horizontal bars the 95% percentiles and dots the minimal and maximal values. Significant differences between treatment groups and the control group are indicated (* p<0.005, ** p <.01; *** p < 0.001).


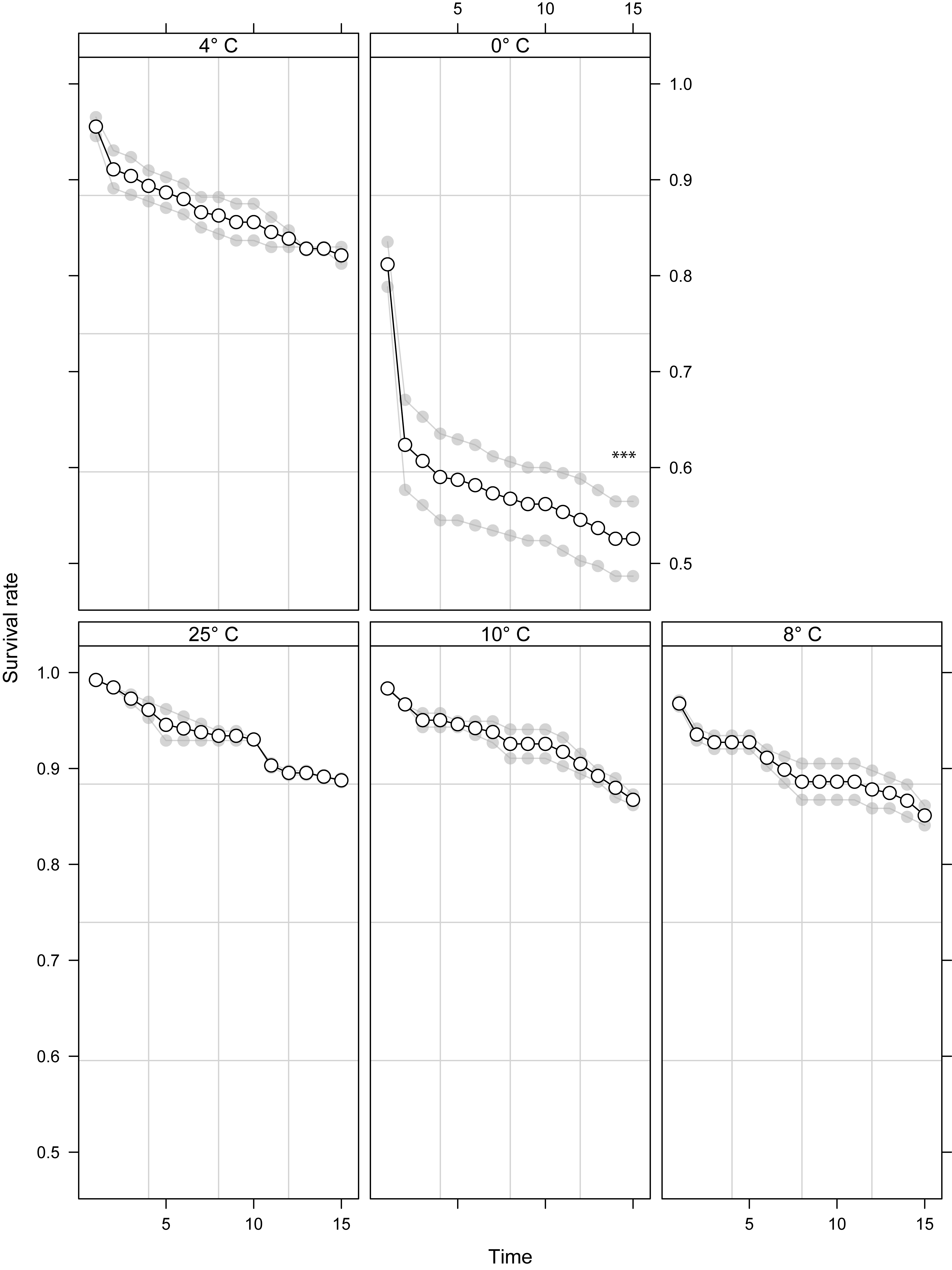


**Fig. S3.** Survival rates of male *Aedes aegypti* exposed to various chilling temperatures over a period of 15 days. Significant differences between treatment groups (10, 8, 4 and 0 °C) and the control group (no chilling - 25°C) are indicated (* p<0.005, ** p <.01; *** p < 0.001).. Individual values of the repeats are indicated in light grey and mean values as a solid line.


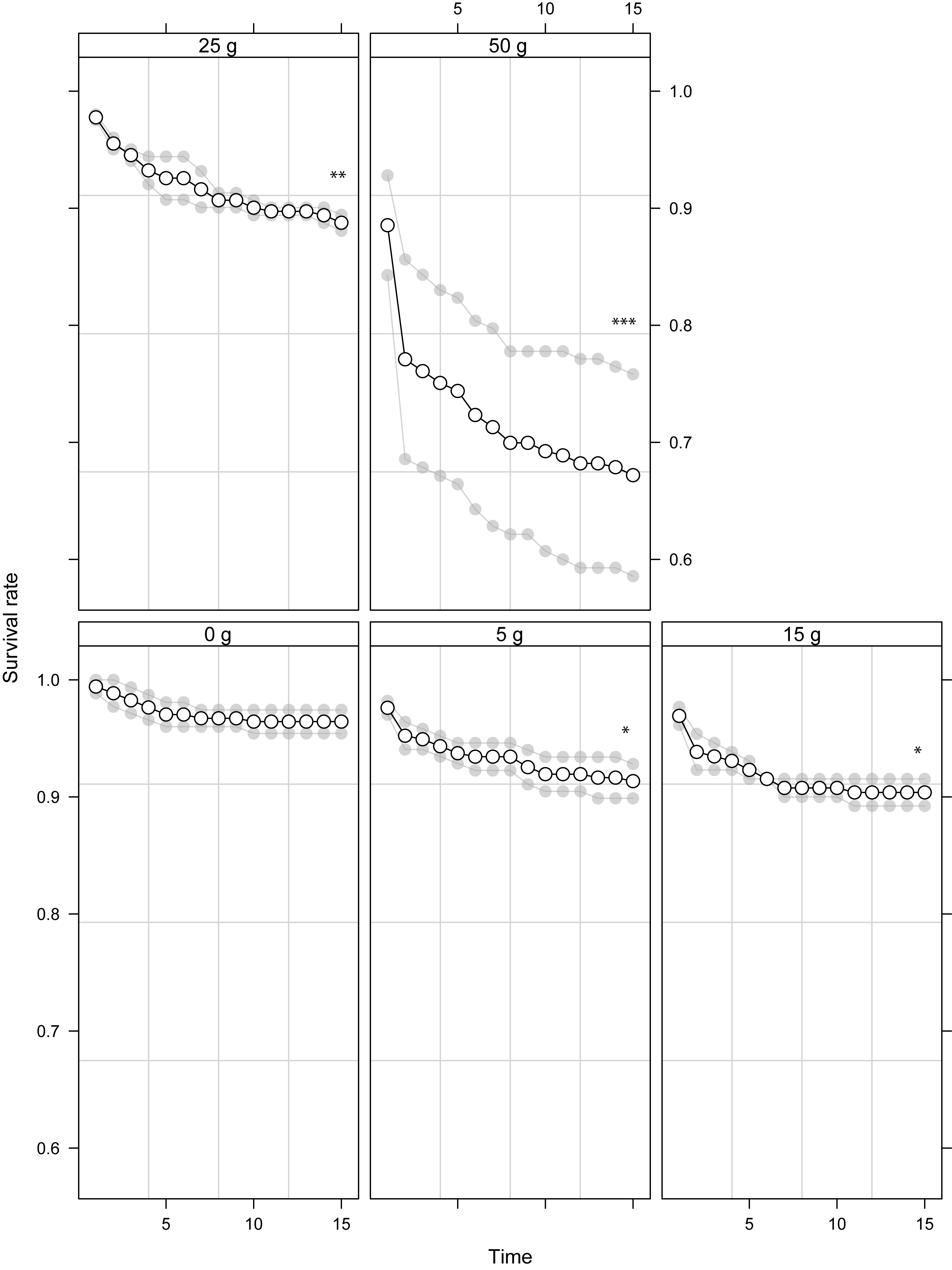


**Fig. S4.** Survival rates of male *Aedes aegypti* exposed to various levels of compaction over a period of 15 days. Significant differences between treatment groups (5, 15, 25 and 50 g) and the control group (no compaction – 0 g) are indicated (* p<0.005, ** p <.01; *** p < 0.001).. Individual values of the repeats are indicated in light grey and mean values as a solid line.

**SI Tables:**

Table S1. Fixed-effects coefficients of a mixed-effect binomial model of the impact

of irradiation dose on survival in *Aedes aegypti* (10 observations, 2 repeats, 6 degrees of freedom)*.*

| Fixed effects | Value | Std. Error | z-value | p-value |
| --- | --- | --- | --- | --- |
| Intercept | 2.5132 | 0.2233 | 11.254 | 2e-16 |
| 30 Gy | -0.1465 | 0.2747 | -0.533 | 0.591 |
| 90 Gy | -2.3992 | 0.2256 | -10.635 | 2e-16 |
| 110 Gy | -2.5056 | 0.2273 | -11.023 | 2e-16 |
| 150 Gy | -2.9113 | 0.2414 | -12.060 | 2e-16 |

Table S2. Fixed-effects coefficients of a mixed-effect binomial model of the impact

of irradiation dose on survival in *Aedes albopictus* (10 observations, 2 repeats, 6 degrees of freedom)*.*

| Fixed effects | Value | Std. Error | z-value | p-value |
| --- | --- | --- | --- | --- |
| Intercept | 2.5200 | 0.2778 | 9.071 | 2e-16 |
| 20 Gy | -0.2820 | 0.3511 | -0.803 | 0.422 |
| 40 Gy | -1.4688 | 0.3097 | -4.742 | 2.11e-06 |
| 80 Gy | -3.5748 | 0.3105 | -11.511 | 2e-16 |
| 100 Gy | -3.7955 | 0.3442 | -11.029 | 2e-16 |

Table S3. Fixed-effects coefficients of a mixed-effect binomial model of the impact

of irradiation dose on full insemination rate in *Aedes aegypti* (25 observations, 5 repeats, , 6 degrees of freedom)*.*

| Fixed effects | Value | Std. Error | z-value | p-value |
| --- | --- | --- | --- | --- |
| Intercept | 2.7300 | 0.5959 | 4.582 | 4.62e-06 |
| 30 Gy | -0.2877 | 0.7917 | -0.363 | 0.716331 |
| 90 Gy | -2.3072 | 0.6650 | -3.470 | 0.000521 |
| 110 Gy | -4.0110 | 0.6949 | -5.772 | 7.82e-09 |
| 150 Gy | -4.8818 | 0.7605 | -6.419 | 1.37e-10 |

Table S4. Fixed-effects coefficients of a mixed-effect binomial model of the impact of irradiation dose on full insemination rate in *Aedes albopictus* (25 observations, 5 repeats, , 6 degrees of freedom)*.*

| Fixed effects | Value | Std. Error | z-value | p-value |
| --- | --- | --- | --- | --- |
| Intercept | 1.9700 | 0.4587 | 4.294 | 1.75e-05 |
| 20 Gy | -1.3346 | 0.5393 | -2.475 | 0.0133 |
| 40 Gy | -3.0097 | 0.5526 | -5.446 | 5.14e-08 |
| 80 Gy | -3.7203 | 0.6107 | -6.092 | 1.11e-09 |
| 100 Gy | -5.8583 | 1.1080 | -5.287 | 1.24e-07 |

Table S5. Fixed-effects coefficients of a mixed-effect binomial model of the impact

of irradiation dose on insemination rate in *Aedes albopictus* (25 observations, 5 repeats, , 6 degrees of freedom)*.*

| Fixed effects | Value | Std. Error | z-value | p-value |
| --- | --- | --- | --- | --- |
| Intercept | 3.1355 | 0.7223 | 4.341 | 1.42e-05 |
| 20 Gy | -0.4729 | 0.9372 | -0.505 | 0.6138 |
| 40 Gy | -2.4116 | 0.7839 | -3.076 | 0.0021 |
| 80 Gy | -3.3098 | 0.7806 | -4.240 | 2.23e-05 |
| 100 Gy | -4.1259 | 0.7920 | -5.210 | 1.89e-07 |

Table S6. Fixed-effects coefficients of a mixed-effect binomial model of the impact of chilling temperature on survival in *Aedes aegypti* (10 observations, 2 repeats, 6 degrees of freedom)*.*

| Fixed effects | Value | Std. Error | z-value | p-value |
| --- | --- | --- | --- | --- |
| Intercept | 2.1379 | 0.2035 | 10.507 | 2e-16 |
| 0 °C | -1.5362 | 0.2376 | -6.467 | 1e-10 |
| 4 °C | -0.3250 | 0.2669 | -1.218 | 0.223 |
| 8 °C | -0.1439 | 0.2839 | -0.507 | 0.612 |
| 10 °C | -0.1278 | 0.2862 | -0.446 | 0.655 |

Table S7. Fixed-effects coefficients of a mixed-effect binomial model of the impact

of chilling temperature on full insemination rate in *Aedes aegypti* (25 observations, 5 repeats, 6 degrees of freedom)*.*

| Fixed effects | Value | Std. Error | z-value | p-value |
| --- | --- | --- | --- | --- |
| Intercept | 2.6813 | 0.6348 | 4.224 | 2.40e-05 |
| 0 °C | -4.7689 | 0.7901 | -6.036 | 1.58e-09 |
| 4 °C | -4.3281 | 0.7340 | -5.896 | 3.71e-09 |
| 8 °C | -1.7908 | 0.6905 | -2.593 | 0.0095 |
| 10 °C | -0.2607 | 0.7988 | -0.326 | 0.7442 |

Table S8. Fixed-effects coefficients of a mixed-effect binomial model of the impact

of compaction on survival in *Aedes aegypti* (10 observations, 2 repeats, 6 degrees of freedom)*.*

| Fixed effects | Value | Std. Error | z-value | p-value |
| --- | --- | --- | --- | --- |
| Intercept | 3.4639 | 0.3279 | 10.565 | 2e-16 |
| 5 g | -0.7809 | 0.3924 | -1.990 | 0.04662 |
| 15 g | -0.8333 | 0.4077 | -2.044 | 0.04095 |
| 25 g | -1.1701 | 0.3774 | -3.101 | 0.00193 |
| 50 g | -2.3055 | 0.3527 | -6.537 | 6.28e-11 |

Table S9. Fixed-effects coefficients of a mixed-effect Binomial model of the impact

of compaction on full insemination rate in *Aedes aegypti* (25 observations, 5 repeats, 6 degrees of freedom)*.*

| Fixed effects | Value | Std. Error | z-value | p-value |
| --- | --- | --- | --- | --- |
| Intercept | 1.6917 | 0.4113 | 4.113 | 3.91e-05 |
| 5 g | -0.8650 | 0.5214 | -1.659 | 0.097114 |
| 15 g | -1.9430 | 0.5038 | -3.857 | 0.000115 |
| 25 g | -2.3848 | 0.5128 | -4.651 | 3.30e-06 |
| 50 g | -4.3997 | 0.7244 | -6.074 | 1.25e-09 |

Table S10. Fixed-effects coefficients of a mixed-effect binomial model of the impact

of irradiation dose on the escape rate from the flight organ in *Aedes albopictus* (10 observations, 2 repeats, 6 degrees of freedom)*.*

| Fixed effects | Value | Std. Error | z-value | p-value |
| --- | --- | --- | --- | --- |
| Intercept | 1.1451 | 0.1941 | 5.901 | 3.62e-09 |
| 20 Gy | -0.3039 | 0.2610 | -1.165 | 0.24420 |
| 40 Gy | -0.6813 | 0.2537 | -2.685 | 0.00724 |
| 80 Gy | -1.2334 | 0.2591 | -4.760 | 1.93e-06 |
| 100 Gy | -2.0553 | 0.2831 | -7.259 | 3.89e-13 |

Table S11. Fixed-effects coefficients of a mixed-effect binomial model of the impact of irradiation dose on the escape rate from the flight organ in *Aedes aegypti* (10 observations, 2 repeats, 6 degrees of freedom).

| Fixed effects | Value | Std. Error | z-value | p-value |
| --- | --- | --- | --- | --- |
| Intercept | 1.94591 | 0.29650 | 6.563 | 5.28e-11 |
| 30 Gy | -0.09633 | 0.41301 | -0.233 | 0.815573 |
| 90 Gy | -0.95266 | 0.36550 | -2.606 | 0.009149 |
| 110 Gy | -1.07949 | 0.35539 | -3.037 | 0.002386 |
| 150 Gy | -1.15745 | 0.34675 | -3.338 | 0.000844 |

Table S12. Fixed-effects coefficients of a mixed-effect binomial model of the impact of chilling temperature on the escape rate from the flight organ in *Aedes aegypti* (10 observations, 2 repeats, 6 degrees of freedom)*.*

| Fixed effects | Value | Std. Error | z-value | p-value |
| --- | --- | --- | --- | --- |
| Intercept | 1.7723 | 0.2121 | 8.355 | 2e-16 |
| 0 °C | -0.1790 | 0.3052 | -0.587 | 0.55743 |
| 4 °C | -0.5881 | 0.2772 | -2.121 | 0.03389 |
| 8 °C | -0.7465 | 0.2842 | -2.627 | 0.00861 |
| 10 °C | -4.2738 | 0.3422 | -12.488 | 2e-16 |

Table S13. Fixed-effects coefficients of a mixed-effect binomial model of the impact

of compaction on the escape rate from the flight organ in *Aedes aegypti* (10 observations, 2 repeats, 6 degrees of freedom)*.*

| Fixed effects | Value | Std. Error | z-value | p-value |
| --- | --- | --- | --- | --- |
| Intercept | 1.2797 | 0.2076 | 6.164 | 7.08e-10 |
| 5 g | -0.5220 | 0.2602 | -2.006 | 0.0449 |
| 15 g | -1.0462 | 0.2421 | -4.322 | 1.55e-05 |
| 25 g | -1.2934 | 0.2360 | -5.480 | 4.25e-08 |
| 50 g | -1.6801 | 0.2446 | -6.868 | 6.52e-12 |
